# Understanding Filipino Rice Farmer Preference Heterogeneity for Varietal Trait Improvements: A Latent Class Analysis

**DOI:** 10.1111/1477-9552.12392

**Published:** 2020-07-14

**Authors:** Rio Maligalig, Matty Demont, Wendy J. Umberger, Alexandra Peralta

**Keywords:** Experimental investment game, latent class cluster analysis, Philippines, preference heterogeneity, rice, varietal trait improvement

## Abstract

Using an experimental methodology based on investment games, we examine whether smallholder rice farmers from Nueva Ecija, Philippines have heterogeneous preferences for improvements in 10 rice varietal traits. We use a latent class cluster approach to identify different segments of rice producing households and their distinct preferences for trait improvements. These clusters were characterised *post hoc* using household, farm, and marketing characteristics. On average, farmers invested the most in rice varietal trait improvements that offered opportunities to reduce losses caused by lodging, insects and diseases. We found four classes of farmers with distinct preferences for improvements in variety traits. The clusters were significantly different in terms of household and farm characteristics. These findings can guide breeding research in the development of varieties that have the traits farmers identified for improvement, and that will address the unique needs of distinct farmer segments.

## 1 Introduction

Rice genetic enhancement research has produced a wide range of improved modern rice varieties. These varieties paved the way for the dramatic increases in rice production seen in many developing countries from the 1960s to 2000s. During this period, Asian rice paddy production more than tripled, increasing from approximately 216 million tons in 1961 to 687 million tons in 2008 (IRRI, 2017). In fact, rice crop improvement research has generated the largest documented impacts seen from investment in agricultural research, accounting for 86% of the total documented impacts in Southeast Asia (Maredia and Raitzer, 2012). Annual gains from adoption of modern varieties in South and Southeast Asia are estimated at US$10.8 billion from the late 1960s to late 1990s (Hossain *et al*., 2003).

In the Philippines, more than 200 inbred varieties and around 80 hybrids were released from the mid‐1960s to 2016 (NSIC, 2016). Despite the numerous varieties released, few have been planted in the field.^1^ For example, Launio *et al*. (2008) used surveys of farm households in major rice producing provinces in the Philippines covering the 1992–1993, 1996–1997 and 2001–2002 crop years. These researchers found that around 70–80% of the surveyed rice areas were planted to only 10 different varieties in one season.^2^ The same trend was reported by Laborte *et al*. (2015) using farm household surveys conducted from 1966 to 2012 in six provinces collectively referred to as Central Luzon. The authors found that less than 10 varieties were planted to 75% of the total rice area in the study sites.^3^

Considering the characteristics of the few varieties adopted by Filipino farmers in the field, it seems that farmers generally place more value on rice varieties with the following traits: high yield, good grain quality, and biotic stress tolerance (resistance to pests and diseases). While these traits appear to be most important to farmers, previous literature suggests that farmer preferences for variety traits are heterogeneous (Birol *et al*., 2012; Ward *et al*., 2014; Kassie *et al*., 2017). This is because farmers are often diverse in terms of their socio‐economic characteristics, household and farm assets, behaviour, experience and attitudes. Furthermore, rice farmers also operate facing different production and marketing systems (Dawe *et al*., 2006; Briones and Dela Peña, 2015).

### 1.1 Objectives

An improved understanding of rice farmers’ unique preferences for varietal traits, and identification of the specific traits that farmers would prefer to see improved, would help breeders prioritise and focus research and, thus, increase subsequent adoption rates in the field. Previous research has shown that farmers are able to identify and suggest improvements in the technologies they use in order to make them more suited for their individual needs (Pingali *et al*., 2001). Therefore, we focus on trait improvements with the practical aim of providing information to breeders and donors to guide research priority setting, resource allocation and the development of product profiles. We argue that this can improve adoption rates for new varieties which better reflect farmers’ needs.

Our main objectives are twofold: (i) to explore the heterogeneity in preferences for rice varietal trait improvements (VTIs); (ii) to examine the factors that contribute to farmer preferences for VTIs. A relatively new cluster analysis method, Latent Class (LC) Cluster Analysis, was used to identify distinct segments of farmers, which differed in their preferences for VTIs as well as their household characteristics. Previous studies have used LC cluster analysis and found evidence of unique segments of farmers having different preferences and attitudes (see Schlecht and Spiller, 2012; Umberger *et al*., 2015; Ochieng and Hobbs, 2016). These previous studies recognised the importance of accounting for heterogeneity in farmer preferences when developing and targeting tailored agricultural policies and programmes. We hope to provide guidance for breeding research in the development of varieties that have traits farmers identified for improvement and that will address distinct farmer segments and needs. We also add to the literature using empirical applications of the LC cluster approach, specifically those addressing agricultural issues at the farmer/producer level.

### 1.2 Investment game approach for eliciting farmer preferences

Farmer preferences for *improvements* in varietal traits (referred to as VTIs) were elicited using an experimental methodology based on the investment game literature (Berg *et al*., 1995; Gneezy *et al*., 2000; Ortmann *et al*., 2000; Cochard *et al*., 2004; Buchan *et al*., 2008; Ahmed, 2011). Drawing on the relevant literature, a novel Investment Game Application (IGA) has been developed by the International Rice Research Institute (IRRI) to elicit preferences, cardinal rankings and values for rice VTIs relative to a current variety (see Demont *et al*., 2015). We use this method rather than other valuation methods (e.g. choice experiment, contingent valuation) because (i) it reflects to the farmers the costs and risks involved in breeding research to improve variety traits, and the resource constraints within which the breeding programmes have to operate, and hence, (ii) it provides the producer’s view of cardinal rankings and relative values of the trait improvement, relative to their nominated replacement variety.

The IGA features a flexible fixed‐cost quadratic (FFCQ) cost function modeling the breeding costs involved in improving the VTI for each trait from the baseline up to the target level for all combinations of VTIs (Demont and Villanueva, 2019). An endowment fund of 100 Philippines pesos (PHP hereafter)^4^ is provided to our farmer participants, representing a share of a one million USD investment in a public rice breeding programme for the development of an improved variety. When farmers invest in traits by increasing the corresponding VTI levels, this fund is proportionally consumed following the FFCQ function. This budget constraint requires our farmer participants to prioritise their investments. The IGA also features a risk function, which models the probability of success of the rice breeding programme in achieving each VTI level and a return function, which models the returns in terms of the societal welfare a particular VTI level generates. The VTIs, cost, return and risk functions were identified and estimated through a series of ‘framed expert elicitation experiments’ with breeders from IRRI and scientists from the National Agricultural Research Systems (NARS) conducted between November 2014 and March 2015 (Demont and Villanueva, 2019).

Because of the flexible fixed‐cost nature of the FFCQ cost function in the IGA, the initial improvement of a trait (from the baseline to the first increment of 5% in the VTI) is more expensive compared to succeeding levels due to fixed start‐up costs such as the establishment of new laboratory or field experiments. Further improvements incur lower costs, reflecting variable incremental costs, which exponentially increase over increasing VTI levels due to the convex nature of the FFCQ cost function. The cost function correctly captures economies of scale and scope in rice breeding; combined costs of certain VTI pairs are sub‐additive, while others are super‐additive (Demont and Villanueva, 2019). For further details on the technical aspects of the IGA, see the online supplementary material (Maligalig *et al*., 2019, Online Appendix A).

Similar to a common investment game where there are two players – a sender and a receiver – the investment game in this study also involved a sender, the farmer participant, and a receiver, IRRI. Farmers decide how much of their endowment fund to send to the receiver, indicating how much they think should be invested in public breeding research for a given trait, which might be thought of as an indication of trusting behaviour (Berg *et al*., 1995; Kocher *et al*., 2015). In the game, IRRI returns a stochastic pay‐off (return to investment) to the farmer depending on the portfolio of VTIs selected. Essentially, IRRI returns the pay‐off to the farmers to maintain the sender’s trust or reciprocate the sender’s trusting behaviour (Berg *et al*., 1995).

The farmer’s stochastic return to his/her investment portfolio (composed of selected VTIs) is subject to the risk incurred by public breeding research programmes in achieving the selected VTIs. These returns represent the social returns that the improved variety – as designed by the farmer in terms of VTIs relative to a replacement variety – would generate if it was released and adopted by farmers after about 6–10 years, assuming that accelerated plant breeding methods were used (Collard *et al*., 2017; Lenaerts *et al*., 2018; Lenaerts *et al*., 2019). However, in this study, breeding investment was framed as an investment with instantaneous return; the returns were calculated in real time and returned to the participant immediately after playing the game.

Farmer participants were given an endowment fund of PHP 100 for the Investment Game, and were first asked to identify their replacement variety (for the variety that they were currently planting) and then asked to indicate the traits of this variety that they wanted to have improved. They were then asked to invest in the VTIs they preferred. The experiment was conducted in Nueva Ecija, a major rice producing province in the Philippines, with 122 rice farming households as the participants.^5^

The IGA methodology used in this study can be considered efficient as a valuation method as it allowed us to incorporate multiple choices for different VTIs, costs of breeding, and the risk of achieving the desired trait improvement. Farmers’ preferred trait improvements will then translate into a corresponding investment portfolio that can guide breeding research in improving variety traits.

The remainder of this paper is organised as follows. In section 2, the relevant literature on adoption and LC cluster analysis used to account for farmer preference heterogeneity is briefly summarised. Section 3 provides an overview of the study sites, sampling strategy and experimental procedures used to elicit farmer preferences and values for VTIs. We also discuss the empirical approach employed to analyse the experimental data. Section 4 reports the findings from the analysis of the experimental data and discusses the results. Section 5 provides the conclusions.

## 2 Analysing Preference Heterogeneity in Technology Traits

### 2.1 Technology adoption and farmer preferences for variety attributes

Our approach, as with other studies of varietal preference (e.g. Smale *et al*., 2001; Lunduka *et al*., 2012; Waldman *et al*., 2016) is based conceptually on Lancaster’s theory of consumer choice (Lancaster, 1966). In examining farmer preferences for variety attributes, it is also important to take into account possible heterogeneity in the preferences (Kline and Wichelns, 1998) which may vary with differences in socio‐economic characteristics, environment, attitudes and tastes.

Differences in preferences have implications in the development of policies and programmes to suit different production and marketing systems (Ouma *et al*., 2007). Ward *et al*. (2014), using a choice experiment, found heterogeneity in preferences for drought tolerant rice varieties among farmers in Bihar, India, where preferences were influenced by whether the drought tolerant trait was expressed in a hybrid or inbred variety.

Kassie *et al*. (2017) also examined preferences for the drought tolerant trait in maize. They found that the heterogeneity in preferences for drought tolerant maize among farmers in Zimbabwe was influenced by gender and occupation of the household head, and by the household size. Birol *et al*. (2012) examined farmers’ preferences for genetically modified (GM) maize using a choice experiment. Using the results of the choice experiment, the authors grouped maize farmers from the Philippines into two groups: the ‘reluctant GM maize farmers’ and the ‘willing GM maize farmers’. They suggested developing policies focused on targeting the needs of the two different segments of farmers. For example, policies related to marketing and extension of GM maize varieties should be targeted to those who were most willing to pay for the GM attribute.

### 2.2 Accounting for preference heterogeneity

Farms in the Philippines are diverse; while many farmers operate small subsistence farms there are also farmers operating larger farms (Koirala *et al*., 2016). Moreover, rice production systems vary considerably due to differences in soil, climate, and economic development conditions (Dawe *et al*., 2006). As a consequence, preferences for varieties and variety traits may also be expected to differ across individual farmers.

Increasingly, Latent Class (LC) cluster analysis (Vermunt and Magidson, 2002) is being used to explore and better identify variations in preferences. We used this clustering technique to identify clusters or segments of farmers with distinct preferences for variety trait improvements. The premise in LC cluster analysis is that there exist unobservable or latent segments of individuals and that an individual belongs to a particular segment. LC cluster analysis is a model‐based clustering approach, where individuals’ class membership probabilities are computed from their observed preferences and from estimated model parameters (Vermunt and Magidson, 2002). In addition, observable variables with mixed scale types (nominal, ordinal, continuous, and counts) and covariates can be used to predict class membership (Vermunt and Magidson, 2003). Previous examples of LC cluster analysis of heterogeneity of farmer preferences include Schlecht and Spiller (2012), Umberger *et al*. (2015) and Ochieng and Hobbs (2016).

## 3 Experimental Approach and Analysis

### 3.1 Ethics approval

The University of Adelaide’s Human Research Ethics Committee approved the study protocols and all data collection instruments (Ethics Approval Number H‐2016‐010). Written informed consent was obtained from all individual participants prior to the actual experiment.

### 3.2 Sampling

#### 3.2.1 Study sites

We purposively selected Nueva Ecija as the study site, since it is a predominantly irrigated major rice producing province in the Philippines, which allowed us to capture farmers’ preferences for VTIs in both wet and dry seasons. Our sample consists of 122 rice producing households, with both the male and female heads of the households participating.

#### 3.2.2 Sampling approach

In the first stage of our multi‐stage sampling approach, we purposely selected three municipalities: Muñoz, Talavera and Guimba. In the second stage, we randomly selected four villages in each municipality. In the final stage, we randomly selected 10 households per village.

Several steps were carried out in the random selection of the villages and rice producing households. First, we approached the Municipal Agriculture Office (MAO) in each of the municipalities to obtain a master list of rice farming households. The master lists include information on the names of the farmers and their respective rice areas classified as irrigated or rainfed. Second, we approached the local officials of the villages selected and asked them to check and verify the names included in the master list. This was done to determine who among the list met the screening criteria for participant selection. The screening criteria were as follows: (i) both the male and female head of the household (husband and wife)^6^ should be involved in rice production or marketing activities; (ii) the household is planting rice in both the wet and dry seasons; and (iii) the household is selling a portion of their rice production. Once the list was verified and checked, a new list per village was generated to include only those households that satisfied the selection criteria. We used a spreadsheet programme to randomly select 10 households per village from these lists to be invited to participate in the experiment. We also randomly selected another set of 10 households per village to serve as a back‐up list in case of no‐show at the onset of the experiment.

#### 3.2.3 Recruitment of participants

The randomly selected households were invited through the designated local field coordinators in each of the selected villages. The local field coordinator was a village official in‐charge of the Agriculture Committee in his or her village. The households were invited to participate through a letter, sent 2 weeks before the scheduled experiment, which explained the details of the research, and the schedule of the experiment. Invited households were then reminded of the schedule 2 days before the actual experiment.

### 3.3 Implementation and procedures

The experiment was framed around a hypothetical context wherein a public breeding programme receives a grant from a donor. The ‘grant’ was distributed in small shares among farmer participants in the experiment. As shareholders in the breeding programme, farmer participants were given the opportunity to allocate their share of the grant to alternative breeding programmes focused on improving rice varietal traits, relative to a participant nominated existing variety.

Prior to administering the IGA, farmers were trained first in the methodology of investing with budget constraints by using the ‘Training on Investment Game Application’ (TIGA). In TIGA, farmers invested in their optimal dish by adding to a fixed amount of rice, a vegetable or meat dish, in 10 increments of PHP 5 subject to a budget constraint of PHP 50 (Figure A1, Online Appendix B). The purpose of TIGA was for farmers to get familiarised with the IGA, particularly in terms of the budget constraint involved and the use of a stylus pen to interact with spin buttons on the tactical screen of a tablet. It was important that the participants be given the chance to use the tablet before the actual game as most of them had not used a tablet‐device previously.

Farmers participated in the experiment using a tablet‐based IGA.^7^ The IGA featured 10 VTI bars, each of them representing a trait metric that can be modified from a baseline value up to a target value in 20 steps or increments of 5% (Table 1). The VTIs can be broadly categorised into (i) grain quality traits – slenderness, aroma, stickiness, and head rice recovery; (ii) stress tolerance traits – lodging tolerance, disease resistance, insect resistance, abiotic stress tolerance, and reduction in shattering; and (iii) an agronomic trait – earliness.^8^ In the IGA, farmers selected their preferred traits to be improved by pulling the VTI bars up to the level that they want a particular trait to be improved.^9^ This was done using the up and down spin buttons (Figure A2, Online Appendix B). As participants allocated their funds among trait improvements, the IGA showed both the remainder of their individual budget available, and the likelihood of achieving the trait improvement selected (i.e. the risk involved in the improvement programme).

**Table 1:** Traits and trait‐specific metrics on which the IGA is calibrated

Trait	Metric	Baseline	Target
*Grain quality traits*			
Slenderness	Length/width ratio	2.4	3.2
Stickiness	Amylose content (%)	27%	22%
Aroma	Price premium (%) (market benchmark = 100%)	0%	100%
Head rice recovery	% head rice obtained from a sample of paddy	45%	60%
*Stress tolerance traits*			
Lodging tolerance	Crop losses eliminated (%)	20%	80%
Disease resistance	Crop losses eliminated (%)	50%	90%
Insect resistance	Crop losses eliminated (%)	80%	95%
Abiotic stress tolerance	Crop losses eliminated (%)	0%	90%
Reduction in shattering	Crop losses eliminated (%)	80%	95%
*Agronomic trait*			
Earliness	Number of days the duration is shortened	0	14

The experimental sessions were held in local training and village halls. There were a total of 12 experimental sessions – one for each village selected. The sessions were conducted in February 2016 over the course of 6 days, each day had one in the morning and one in the afternoon. The 12 sessions were divided in four groups of three sessions to accommodate four information treatments used to test whether there would be differences in the preferences of farmers when given access and exposure to particular information. The first information treatment was the control, where no information was provided. The second was the market information treatment, which included information on the most preferred rice traits of urban (Metro Manila) consumers (Custodio *et al.,* 2016). The third treatment was climate change information, including increasing climate variability and the rise in frequency of extreme weather events, which can produce more frequent droughts, floods, and more uncertainty in rainy/wet season onset. The fourth information treatment combined both market information and climate change information. The assignment of the information treatments was randomly drawn prior to the start of all experimental sessions. Each session ran through the following stages: (i) registration, (ii) introduction of the research team, (iii) information treatment, (iv) explanation of the experiment, (v) presentation and explanation of the IGA, replacement variety, and VTIs, (vi) training on the IGA, (vii) six consecutive rounds of IGA, (viii) short post‐experiment survey, and (ix) payment of returns and closure of the session. The sessions were conducted using the local language, Filipino. Details of the experimental procedures are available online (Maligalig *et al*., 2019, Online Appendix A).

After the training and explanation of their tasks, the husband (H) and wife (W) played the IGA for two target seasons, the wet (WS) and the dry (DS) simultaneously and independently (Round A: H/WS; Round B: W/WS; Round C: H/DS; Round D: W/DS). They then played the IGA jointly (J) for two seasons as well (Round E: J/WS; Round F: J/DS). At the start of each round, farmers were asked to identify their replacement (comparator) variety. The facilitator explained to the farmers that the replacement variety was what they would be asked to improve upon to obtain their ideal variety. Participants were told that the replacement variety could be their most preferred variety, or the most popular variety grown in their area. They were told that the replacement variety can be a variety that they were currently growing or they may or may have not grown it in the past. After selecting their preferred replacement variety, farmers were then asked to choose from 10 VTIs to invest in.

During the independent rounds (Rounds A–D), the husband and wife were each assigned an ‘agent’ who facilitated the IGA and the post‐experiment survey. The post‐experiment survey consisted of two parts. The first part included general questions on household, farm and marketing practices. Households completed the first part either before or after being administered the IGA. The second part asked the participants on the motivations behind their allocation decisions in IGA and a short quiz (two questions) to verify how well they had understood the experiment. In the consensus round (Rounds E–F), a different agent with a different tablet was assigned per couple. To provide equal opportunity in answering the IGA during the consensus round, the husband and wife were given separate stylus pens and the tablet was placed in the middle of their table. However, following similar protocols in experimental economics involving collective induction (Demont *et al*., 2013), no further instructions were given, and households were free to decide on how to achieve consensus.

In each round, participants had an available endowment fund amounting to PHP 100 to invest in the VTIs. However, this amount was not given in cash at the beginning of the experiment. Instead, a final stochastic pay‐off, subject to risk, was given at the end of the experiment. If farmers chose not to invest, their initial, risk‐free endowment of PHP 100 was given. On top of the final pay‐off was a fixed show‐up fee amounting to PHP 250 paid to each household. This was equivalent to around 3 hours of paid agricultural labour per participant, corresponding to the average time farmers had to give up for participating in the experiment.^10^

Similar to experimental auction procedures (Demont *et al*., 2013) and to reduce costs, only one round was selected as binding. After all households completed all six rounds (Rounds A–F) of the IGA, the final pay‐off was determined after randomly selecting a ‘binding’ round, by rolling a dice, as is commonly done in experimental economics (e.g. Lusk and Shogren, 2007). Depending on the VTI levels and risk levels associated with each VTI in the investment portfolio, the IGA computed a stochastic return to investment (Demont *et al*., 2015). The resulting cash returns, augmented by the fixed show‐up fee, were placed in an envelope and distributed to the couples one at a time. A single‐blind payment protocol was used where the research team knew the participants’ earnings, but the participants did not know other participants’ earnings. On average, each household earned PHP 1,210, which was equivalent to four daily wages for agricultural labour, with a maximum amount of PHP 2,300. This was on top of the PHP 250 show‐up fee.

### 3.4 Latent class clustering analysis

The LC clustering method was used to identify the existence of distinct segments of rice farming households based on their preferences for the 10 rice VTIs elicited using the IGA. Specifically, households’ investment shares for each of the 10 VTIs elicited during the two joint IGA rounds (Round E: J/WS and Round F: J/DS) were used to represent the participants’ risk informed and budget constrained VTI preferences. Thus, the investment shares/preferences from Round E and Round F were used as ‘indicator variables’ in the LC clustering. The preference ‘indicator variables’ were continuous variables representing households’ joint investment of their endowment fund across the 10 different VTIs during the wet and the dry seasons. It is important to note that only the household‐level decisions (joint husband and wife allocations from Round E and Round F) were used in the LC analysis; gendered differences between joint and individual VTI portfolios and intra‐household decision making were analysed (using different techniques) and are the focus of another study (see Maligalig *et al*., 2019).

Following Vermunt and Magidson (2002), the basic LC cluster model for continuous indicator variables under the assumption of local independence among all indicators and without covariates can be specified as:

(1) $$f\left(y_{i}|\theta \right)=\sum_{k=1}^{K}\pi_{k}f_{k}\left(y_{i}|\theta_{k}\right).$$

In equation (1), $y_{i}$ is a vector of indicator variables^11^ (household joint investment shares), K is the number of clusters, and $\pi_{k}$ denotes the prior probability of belonging to a latent class or cluster k.

Two main methods to estimate LC cluster model parameters are the maximum‐likelihood (ML) method and the maximum‐posterior (MAP) method (Vermunt and Magidson, 2002), where the classification of the indicators into clusters is of particular interest. Using Latent GOLD^®^ 5.1, this classification is done based on assigning each object to the class with the highest posterior class membership probabilities, as shown in equation (2):

(2) $$\pi_{\left(k\right|y_{i}}=\frac{\pi_{k}\prod_{j}f_{k}\left(y_{\mathrm{ij}}|\theta_{\mathrm{jk}}\right)}{\sum_{k}\pi_{k}\prod_{j}f_{k}\left(y_{\mathrm{ij}}|\theta_{\mathrm{jk}}\right)}$$

To identify the optimal number of clusters, the most widely used model selection tools are information criteria such as the Akaike Information Criteria (AIC) and Bayesian Information Criteria (BIC). When comparing models with different numbers of clusters, the lower the value of the AIC or BIC, the better the fit (Vermunt and Magidson, 2005).

Additionally, LC cluster models can also be assessed based on how well the latent classes are separated using the classification likelihood criterion (CLC) and the approximate weight of evidence (AWE). The lower the value of these classification statistics, the better (Vermunt and Magidson, 2016). However, as these formal guides/rules can sometimes be difficult to achieve in practice, the selection of the number of clusters can also be based on the parsimony and interpretability of the model (Swait, 1994).

A basic assumption of the LC model is the local independence assumption, which states that indicators are mutually independent given that an individual belongs to a certain latent class (Vermunt and Magidson, 2016). Vermunt and Magidson (2016) proposed an alternative model fitting strategy, which is to relax the local independence assumption by allowing direct effects or association between indicators that have significantly large bivariate residuals (BVR). In our case, we found BVRs that are significantly larger than one; thus, as proposed by Vermunt and Magidson (2016) we allowed direct association between pairs of indicators that had significantly large BVR.

The final step of the analysis involved a post‐hoc analysis of each segment to examine possible differences among clusters in terms of preferences for VTIs, and household, farm, and marketing characteristics.

## 4 Results and Discussion

### 4.1 Household investment shares in rice VTIs

We found that households invested an average of 99% of their investment shares. Table 2 reports the mean investment shares for all VTIs by cropping season.^12^ Interestingly, there are some statistically significant differences in mean investments across the wet and dry season. In the wet season, the VTI ‘lodging tolerance’ had the highest mean investment share (21% in the wet season versus only 6% in the dry season). However, in the dry season, the VTI ‘insect resistance’ received the highest mean investment share (23% in the dry season versus 19% in the wet season). Mean investment shares for the grain quality traits ‘slenderness’, ‘aroma’ and ‘head rice recovery’ were significantly higher in the dry season than in the wet season. Mean investment in ‘abiotic stress tolerance’ was significantly higher in the wet season compared to the dry season.

**Table 2:** Mean joint investment shares by VTI and cropping season

VTI	Wet season (*n* = 122)				Dry season (*n* = 122)				Mean Diff.	*t*‐value	df
	Mean	Std. Dev.	Min	Max	Mean	Std. Dev.	Min	Max			
*Grain quality traits*											
Slenderness	0.07	0.14	0.00	0.70	0.11	0.17	0.00	0.72	–0.04	2.78^***^	121
Stickiness	0.04	0.11	0.00	0.50	0.04	0.11	0.00	0.55	0.01	–0.44	121
Aroma	0.03	0.09	0.00	0.40	0.05	0.12	0.00	0.45	–0.02	2.20^**^	121
Head rice recovery	0.05	0.13	0.00	0.55	0.10	0.18	0.00	0.65	–0.05	2.95^***^	121
*Stress tolerance traits*											
Lodging tolerance	0.21	0.21	0.00	1.00	0.06	0.13	0.00	0.47	0.15	–7.06^***^	121
Disease resistance	0.15	0.20	0.00	0.63	0.17	0.21	0.00	0.68	–0.02	1.03	121
Insect resistance	0.19	0.20	0.00	1.00	0.23	0.20	0.00	1.00	–0.04	1.89^*^	121
Abiotic stress tolerance	0.11	0.20	0.00	0.80	0.08	0.17	0.00	0.69	0.04	–1.72^*^	121
Reduction in shattering	0.10	0.12	0.00	0.53	0.12	0.14	0.00	0.54	–0.02	1.20	121
*Agronomic trait*											
Earliness	0.04	0.10	0.00	0.45	0.04	0.10	0.00	0.48	0.00	–0.01	121

Notes

Mean difference is wet season investment share minus dry season investment share.

^***^, ^**^, ^*^ denote statistical significance at the 1%, 5%, and 10% levels, respectively, for paired *t*‐test.

### 4.2 LC cluster analysis

Using Latent GOLD^®^ 5.1, we estimated models ranging from one to six clusters. The four‐cluster solution was selected as it has the lowest BIC value and yielded an improved model fit with local dependencies among the indicators (i.e. lower BIC value and classification error).^13^ Thus, the results presented and discussed in detail are based on the four‐cluster model with local dependencies.

### 4.3 Characterisation of the clusters

Breeders often construct product profiles relative to one or two dominant mega‐varieties that need to be replaced. Table 3 suggests that households among all clusters tend to converge towards the same two dominant replacement varieties per season, that is, NSIC Rc222 and NSIC Rc216 in the wet season and SL‐8H and NSIC Rc222 in the dry season. This implies that breeding programmes can confidently consider these varieties as references in their variety replacement programmes.

**Table 3:** Replacement varieties selected by households in each cluster (*n* = 122)

Season/ Replacement variety	Cluster 1		Cluster 2		Cluster 3		Cluster 4		Fisher’s exact test	df
	Stress tolerance focused (*n* = 62)		Mixed‐focus (*n* = 36)		Insect resistance, head rice recovery & earliness focused (*n* = 13)		Grain quality focused (*n* = 11)			
	Freq.	%	Freq.	%	Freq.	%	Freq.	%		
*Wet season*									4.79	6
NSIC Rc222	47^a^	75.81	32 ^a^	88.89	11 ^a^	84.62	10 ^a^	90.91		
NSIC Rc216	11^a^	17.74	3 ^a^	8.33	2 ^a^	15.38	0	0.00		
Others	4^a^	6.45	1 ^a^	2.78	0	0.00	1 ^a^	9.09		
*Dry season*									8.77	6
SL‐8H	43^a^	69.35	21 ^a^	58.33	9 ^a^	69.23	6 ^a^	54.55		
NSIC Rc222	7^a^	11.29	9 ^a,b^	25.00	2^a,b^	15.38	5 ^b^	45.45		
Others	12^a^	19.35	6 ^a^	16.67	2^a^	15.38	0	0.00		

Notes

Using Bonferroni post‐estimation, different superscript letters in each row indicate statistically significant difference in values between columns at the 5% level. Values without superscript were not used in comparisons since their column proportion is equal to zero.

Table 4 summarises the statistics for the rice VTIs for each of the four clusters. Cluster names/labels are also provided, and these labels attempt to characterise each cluster based on the VTI or VTIs with the highest investment share(s) for the cluster. Table 5 shows the results of the *post‐hoc* analysis of the four clusters’ household, farm and marketing characteristics. Respondents were assigned to the cluster with the highest posterior membership probability (Vermunt and Magidson, 2016). In Table 6, we show the mean posterior probability of cluster members belonging to other clusters, which are very small to zero in all cases.

**Table 4:** Mean VTI investment shares by cluster

Season/VTI	Cluster 1	Cluster 2	Cluster 3	Cluster 4	ANOVA test (*p*‐value)
	Stress tolerance focused (*n* = 62)	Mixed‐focus (*n* = 36)	Insect resistance, head rice recovery & earliness focused (*n* = 13)	Grain quality focused (*n* = 11)	
Cluster size (%)	50%	30%	11%	9%	
Wet season					
*Grain quality traits*					
Slenderness	0.03^a^	0.10	0.02^a^	0.25^b^	0.00
Stickiness	0.03	0.03	0.08	0.10	0.13
Aroma	0.00^a^	0.00^a^	0.00^a^	0.31^b^	0.00
Head rice recovery	0.03	0.04	0.17	0.07	0.00
*Stress tolerance traits*					
Lodging tolerance	0.23^a^	0.23^a^	0.18	0.04^b^	0.04
Disease resistance	0.23^a^	0.10^b^	0.05^b,c^	0.00^c^	0.00
Insect resistance	0.21^a^	0.18	0.23^a^	0.06^b^	0.11
Abiotic stress tolerance	0.09	0.18	0.10	0.04	0.11
Reduction in shattering	0.12^a^	0.11	0.08	0.04^b^	0.19
*Agronomic trait*					
Earliness	0.02	0.04	0.08	0.09	0.10
Dry season					
*Grain quality traits*					
Slenderness	0.05^a^	0.16^b^	0.12	0.24^b^	0.00
Stickiness	0.02	0.05	0.00	0.11	0.04
Aroma	0.03^a^	0.04^a^	0.00^a^	0.25^b^	0.00
Head rice recovery	0.05	0.15	0.23	0.06	0.00
*Stress tolerance traits*					
Lodging tolerance	0.12^a^	0.01^b^	0.00^b^	0.00^b^	0.00
Disease resistance	0.33^a^	0.00^b^	0.00^b^	0.06^b^	0.00
Insect resistance	0.23	0.28^a^	0.25	0.09^b^	0.06
Abiotic stress tolerance	0.06	0.12	0.02	0.07	0.24
Reduction in shattering	0.11	0.18^a^	0.08	0.03^b^	0.01
*Agronomic trait*					
Earliness	0.00^a^	0.00^a^	0.29^b^	0.09^a^	0.00

Notes

In each row, different superscript letters indicate statistically significant difference in values between columns at the 5% level (*post‐hoc* Games‐Howell test).

**Table 5:** Means of household, farm, and marketing characteristics

Variable	Description	Cluster 1	Cluster 2	Cluster 3	Cluster 4	Mean	Std. Dev.	*p‐*value ^a^
		Stress tolerance focused	Mixed‐focus	Insect resistance, head rice recovery & earliness focused	Grain quality focused			
Age – husband	Age of husband in years	49.85^a^	48.31^a^	52.15^a^	61.73^b^	50.71	10.59	0.00
Age – wife	Age of wife in years	47.10^a^	44.81^a^	49.31	58.18^b^	47.66	10.75	0.00
Education – husband	Years in school of husband	8.34	8.39	8.54	8.82	8.42	2.60	0.95
Education – wife	Years in school of wife	8.19	8.08	8.62	7.64	8.16	2.39	0.79
Farm experience – husband	Years of farming experience of husband	27.68	23.75	30.31	31.73	27.16	12.00	0.14
Farm experience – wife	Years of farming experience of wife	17.98	16.31	20.69	26.82	18.57	13.81	0.15
Training attendance – husband	1 – attended agricultural training in the past, 0 – otherwise	0.71	0.61	0.85	0.73	0.70	0.46	0.45
Training attendance – wife	1 – attended agricultural training in the past, 0 – otherwise	0.13	0.19	0.31	0.18	0.17	0.38	0.46
Membership in organisation – husband	1 – member of an organisation, 0 – otherwise	0.44	0.50	0.46	0.64	0.48	0.50	0.66
Membership in organisation – wife	1 – member of an organisation, 0 – otherwise	0.37	0.28	0.23	0.64	0.35	0.48	0.13
Time preference – husband	Preference for present values as measured by a discount factor	1.71^a^	0.53^b^	4.54	1.36	1.63	4.67	0.07
Time preference – wife	Preference for present values as measured by a discount factor	1.73	1.00	1.42	0.93	1.41	2.00	0.30
Risk appetite in investing in farming – husband	1 – extremely unlikely, 2 – unlikely, 3 – neutral, 4 – likely, 5 – extremely likely	4.97	4.97	5.00	4.64	4.94	0.32	0.20
Risk appetite in investing in farming– wife	1 – extremely unlikely, 2 – unlikely, 3 – neutral, 4 – likely, 5 – extremely likely	4.77^a^	4.75^a^	4.85	5.00^b^	4.80	0.43	0.60
Past experience – husband	1 – investment preference for VTIs based on past experience, 0 – otherwise	0.65	0.44	0.62	0.82	0.60	0.49	0.10
Past experience – wife	1 – investment preference for VTIs based on past experience, 0 – otherwise	0.45	0.44	0.38	0.36	0.43	0.50	0.93
Household size	Number of household members	4.56	4.53	5.69	4.27	4.65	1.90	0.20
Income	Annual family income in ‘000 PHP	69.10	76.08	74.82	92.83	73.91	52.78	0.58
Farm size	Total landholdings in hectares (own + lease)	1.50^a^	1.22	0.86^b^	0.98	1.30	1.04	0.12
Percent leased area	Proportion of leased area to total landholdings	0.43	0.53	0.31	0.49	0.45	0.49	0.55
Yield	Production per hectare in metric tons	6.26	6.15	6.52	5.81	6.22	1.50	0.69
Distance to market	Distance of the farm to market in km	3.78	5.58	3.08	2.36	4.11	5.71	0.26
Proportion sold	Proportion of total production sold	0.67	0.63	0.57	0.53	0.63	0.19	0.08
Buyers’ standard requirement	1 – buyers require certain quality standards, 0 – otherwise	0.69	0.47	0.69	0.55	0.61	0.49	0.16
Knows the end market	1 – knows the end market for their rice crop, 0 – otherwise	0.29	0.44	0.31	0.45	0.35	0.48	0.40
Market information	1 – exposed to market information treatment, 0 – otherwise	0.40	0.56	0.46	0.82	0.49	0.50	0.06
Climate change information	1 – exposed to climate change information treatment, 0 – otherwise	0.50	0.50	0.54	0.64	0.52	0.50	0.86

Notes

In each row, different superscript letters indicate statistically significant difference in values between columns at the 5% level (*post‐hoc* Games‐Howell test).

^a^ Risk appetite in investing in farming was tested using Fisher’s exact test, all others using ANOVA.

**Table 6:** Mean posterior probability of class membership in each cluster

Class	*N*	1	2	3	4
1	62	0.99	0.01	0.00	0.00
2	36	0.01	0.99	0.00	0.00
3	13	0.00	0.00	1.00	0.00
4	11	0.00	0.00	0.00	1.00

Cluster 1 is the largest segment with 50% of the households (Table 4). Most of the households in Cluster 1 selected NSIC Rc222 in the wet season and SL‐8H in the dry season as their replacement varieties (Table 3). These (dominant) replacement varieties are relatively inferior to other varieties with respect to traits such as tolerance to stress and resistance to pests and diseases. Hence, compared to other clusters, Cluster 1 invested significantly more in lodging tolerance and disease resistance in the wet season. They also had high investment shares in insect resistance and reduction in shattering in both the wet and dry seasons. On the other hand, they invested significantly less in slenderness and aroma in both seasons. This cluster was thus labelled the ‘Stress Tolerance Focused Cluster’. Compared to Cluster 4, the wives in Cluster 1 had a lower score in terms of risk appetite as measured through their willingness to take risks in investment in farming (Table 5).

Cluster 2, which accounts for 30% of the households, had significantly higher investment shares in lodging tolerance in the wet season compared to other clusters, except Cluster 1. Moreover, households in this cluster had significantly higher investments in slenderness, insect resistance, and reduction in shattering in the dry season. This cluster was labelled the ‘Mixed‐focus Cluster’. Households in the ‘Mixed‐focus Cluster’ prioritised these traits to address the poor tolerance to stress and resistance to pests of the dominant replacement varieties NSIC Rc222 and SL‐8H (Table 3). The ‘Mixed‐focus Cluster’ also considered consumer preferences by investing in slenderness, a trait that is lacking in SL‐8H. Husbands in the ‘Mixed‐focus Cluster’ had the lowest discount factor. This may explain why they did not invest in earliness (Table 4); as there may be little ‘urgency’ for them in terms of having an early harvest.

Cluster 3 accounts for 11% of the households (Table 4). Most of the households in Cluster 3 selected NSIC Rc222 as their replacement variety in the wet season (Table 3). To address the limited resistance of NSIC Rc222 to pests, Cluster 3 households invested significantly more in insect resistance in the wet season. In the dry season, the majority of the households in this cluster identified SL‐8H as their replacement variety (Table 3). Although SL‐8H is an early maturing variety, farmers still preferred to shorten the days to maturity by investing more in earliness. In terms of grain quality traits, households in this cluster showed greater investment in head rice recovery in both wet and dry seasons, but invested significantly less in slenderness in the wet season and aroma in both seasons. This cluster was thus labelled as the ‘Insect Resistance, Head Rice Recovery and Earliness Focused Cluster’.

Cluster 4, the smallest cluster with 9% of the households, was labelled as the ‘Grain Quality Focused Cluster’ as they invested around 60–67% of their endowment fund in aroma, slenderness and stickiness combined (Table 4). Their investments in slenderness and aroma were significantly higher than the other clusters.

Most households in the ‘Grain Quality Focused Cluster’ selected NSIC Rc222 in the wet season and SL‐8H in the dry season as their replacement varieties (Table 3). In the dry season, the proportion of Cluster 4 households that identified NSIC Rc222 as their replacement variety was significantly higher than the proportion of Cluster 1 households that also chose NSIC Rc222 as their replacement variety (Table 3). Although NSIC Rc222 already has the grain quality traits that consumers prefer (long and slender), Cluster 4 households invested more in aroma, consistent with the increasing trend in demand for aroma in urban areas in the Philippines (Custodio *et al*., 2016; Custodio *et al*., 2019). NSIC Rc222 is a non‐aromatic variety. In the dry season, Cluster 4 households also invested more in slenderness, a trait that is lacking in the replacement variety SL‐8H.

The couples in the ‘Grain Quality Focused Cluster’ were significantly older (Table 5). Compared to other clusters, the wives had the highest risk appetite in investing in rice farming. Finally, although the difference was only weakly significant at the 6% level, we observe that households who had been treated with our forward‐looking information on market trends were more likely to become part of this cluster.

## 5 Conclusions

We examined heterogeneity in farmer preferences for improvements in rice variety traits using data gathered from experimental investment games conducted in Nueva Ecija, Philippines. A major aim of the investigation was to identify the traits which farmers consider the most important to improve, compared with their selected comparator variety, so as to inform plant breeding programmes. The investment game allowed us to reflect both the relative costs and risks associated with the plant breeding processes in achieving trait improvement to the participants, hence informing their choices.

We used a latent class (LC) cluster analysis to identify different segments of rice producing households and each segment’s distinct preferences. The identified clusters were characterised *post hoc* using household, farm and marketing characteristics. The results suggest that accounting for heterogeneity in preferences is important as farmers have different socio‐economic characteristics, which appear to influence their investments in, and hence preferences for VTIs.

The LC cluster analysis revealed four segments of rice farming households, each with unique preferences for VTIs. This is in line with previous studies that have identified different segments of farmers based on their preferences for variety attributes (Dalton *et al*., 2011; Birol *et al*., 2012). The identified clusters were: stress tolerance focused (50%), mixed‐focus (30%), insect resistance, head rice recovery and earliness focused (11%), and grain quality focused (9%).

Although all clusters had the same two dominant replacement varieties per season (i.e. NSIC Rc222 and NSIC Rc216 in the wet season and SL‐8H and NSIC Rc222 in the dry season), each cluster had different priorities for variety traits that they preferred to be improved. The stress tolerance focused cluster prioritised lodging tolerance and disease and insect resistance, while the grain quality focused cluster invested more in slenderness and aroma. The mixed‐focus cluster had higher investment shares in lodging tolerance, slenderness, insect resistance and reduction in shattering, while Cluster 3 prioritised insect resistance, head rice recovery and earliness.

The results suggest that relying on averages or means of investment shares for each VTI for each replacement variety and season would overlook the other VTIs that were prioritised by some clusters. Therefore, in terms of product profiles that breeders can use in their breeding priorities and decisions, our results imply that more than one product profile could be developed for each replacement variety considering the heterogeneous preferences of farmers for VTIs. This may then lead to the development of two or more improved varieties to address the diverse sets of preferences or just one improved variety that would include most of the preferred VTIs. Depending on the available resources, breeders can use these results to develop a portfolio of improved varieties for two distinct growing seasons to serve up to four distinct farmer clusters. The development of portfolios of improved varieties with traits that address the unique needs of heterogeneous rice farmers may lead to increased adoption rates and, ultimately, improvements in the welfare of smallholder rice farmers as a result of increases in on‐farm productivity and profitability.

Although we found four unique segments of rice farming households, there are limitations in terms of the generalisation of the findings to a larger population of farmers. This is because our sample selection is limited to just one province in the Philippines. Farmers in other areas or provinces would also have distinct profiles and operate on different production and marketing systems. As such, it is recommended that similar research is done on other rice producing provinces to identify further differences in preferences, which can help in the development of rice varieties that are better suited to the unique preferences and needs of the farmers.

## Supporting information

Supplementary Material

Supplementary Material

## Footnotes

^1^ The low adoption of released varieties can be explained by both demand and supply issues. The supply issues are reviewed elsewhere (e.g. Mataia *et al.,* 2011; Mariano *et al.,* 2012); here, we take a closer look at farm‐level demand issues.

^2^ The varieties adopted were generally high yielding, with good grain qualities, resistant to pests and diseases, and early maturing. Some of the varieties (e.g. IR64, IR74, IR42) adopted during these periods were released back in the 1970s and 1980s.

^3^ The varieties adopted were high yielding, early maturing, with long and slender grains, high milling recovery, and intermediate amylose content.

^4^ At the time of the experiment (February 2016), one US dollar was equivalent to approximately PHP 48.

^5^ Resource constraints prevented a larger sample in this case. Pre‐testing of the IGA experiment was conducted in Victoria, Laguna, Philippines in May 2015. The session took two hours to finish (Demont *et al*., 2015). The IGA experiment was then conducted in Eastern India in October 2015 (Ynion et al., 2015), in Bangladesh in September–October 2016 (Ynion et al., 2016), and in Cambodia in September 2018 (IRRI, 2018).

^6^ We included this criterion to collect gender‐disaggregated data and study gendered preferences and intra‐household decision making in another study (see Maligalig *et al.,* 2019).

^7^ The application was written in Microsoft Excel 2010 and designed to run on Windows 8 computer tablets.

^8^ As yield is what breeders call a ‘must trait’, it was not included as a specific varietal attribute in the IGA. However, as farmers would likely be aware, ‘stress tolerance’ traits and the ‘agronomic’ trait will affect yield.

^9^ During the experimental sessions, we provided detailed explanations of each varietal trait improvement using simple language and visual aids. The visual aids were available to farmers for clarification during the experimental sessions (see Maligalig *et al.,* 2019, Online Appendix A).

^10^ At the time of the experiment, the minimum daily wage rate for agricultural labour in the province was PHP 334 (PSA, 2016).

^11^ Alternative labels for the y variables are dependent variables, outcome variables, outputs, endogenous variables, or items.

^12^ A reviewer interestingly pointed out that consumer or end‐market demand for certain individual VTIs may be correlated. For example, demand in Southeast Asia is converging to the characteristics of Jasmine rice (Custodio *et al.,* 2019; Custodio *et al.,* 2016), which is typically aromatic, soft, somewhat sticky and slender. However, in the IGA, demand for VTIs depends on the limitations of the replacement variety that farmers propose in the first place. For example, it is well known that NSIC Rc222 lacks stickiness, but is sufficiently slender. As a result, we see that most farmers who choose this replacement variety invest in stickiness, but few invest in slenderness. Furthermore, farmers’ demand for individual traits (e.g. traits such as insect resistance) may not be the same as the traits that consumers demand (e.g. quality traits).

^13^ From discussions with rice breeders, we also learned that four distinct product profiles per region would represent about the appropriate level of granularity to target in their variety replacement programmes, given the available resources.
