# Supplementary material for: Understanding Filipino Rice Farmer Preference Heterogeneity for Varietal Trait Improvements: A Latent Class Analysis
Source: J Agric Econ. 2020 Jul 14;72(1):134–57. doi: 10.1111/1477-9552.12392 (PMC7818485; doi:10.1111/1477-9552.12392)
Supplement: Supplementary file 2 — Supplementary Material [file JAGE-72-134-s002.docx]

**Understanding Filipino Rice Farmer Preference Heterogeneity for Varietal Trait Improvements: A Latent Class Analysis**

Rio Maligalig, Matty Demont, Wendy J. Umberger and Alexandra Peralta

**Online Appendix B**


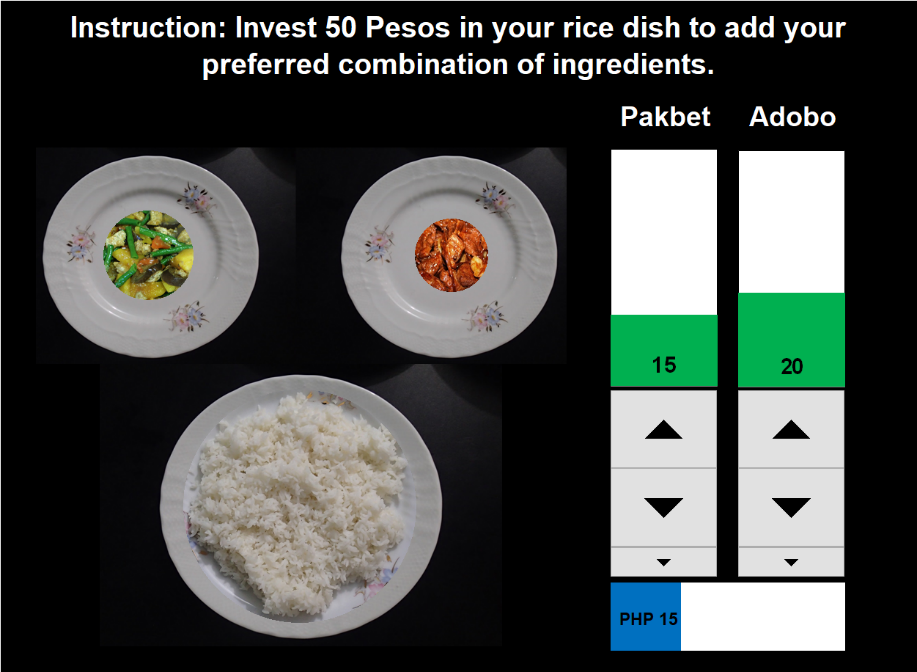


Figure A1. Training on Investment Game Application (TIGA) with example allocation of PHP 15 to Pakbet (vegetable dish) and PHP 20 to adobo (meat dish). The blue horizontal bar at the bottom shows the remaining budget of the initial PHP 50 endowment fund.

**
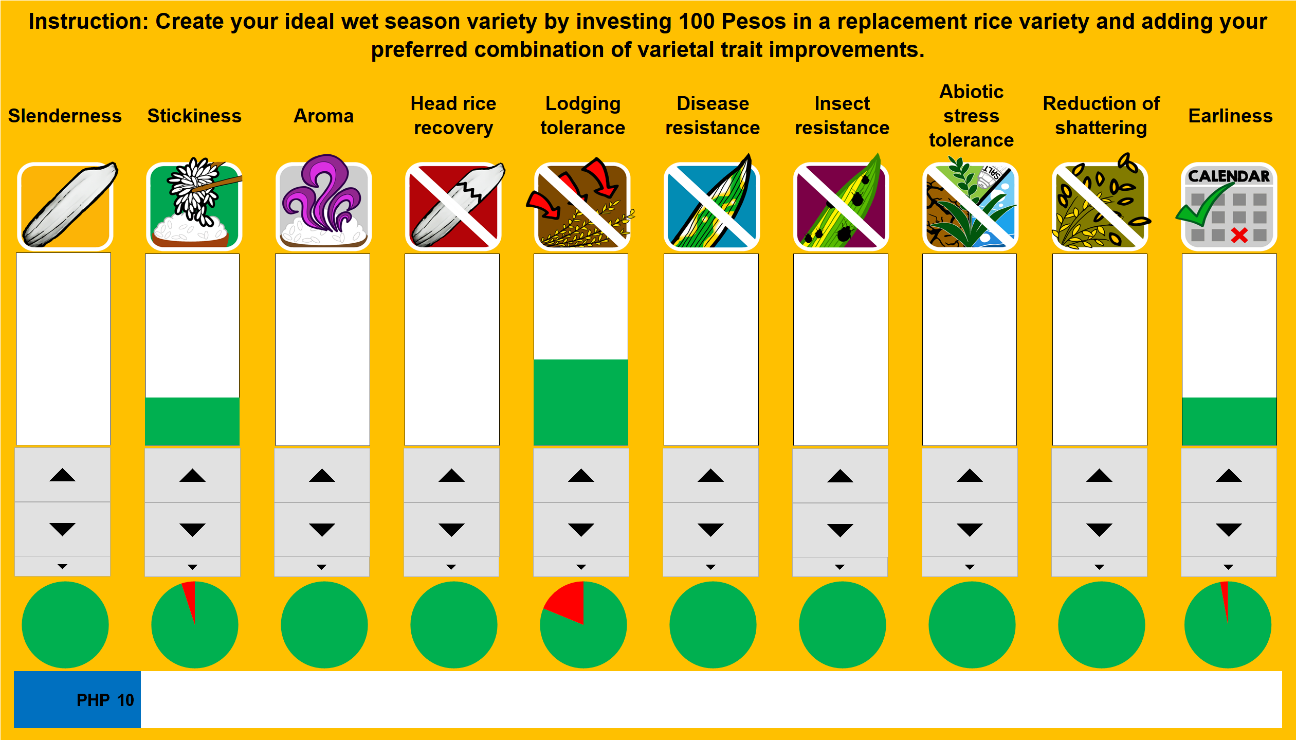
**

Figure A2. Investment Game Application (IGA) with example allocations in stickiness, lodging tolerance, and earliness. The blue horizontal bar at the bottom shows the remaining budget of the initial PHP 100 endowment fund, while the pie charts below the VTI bars indicate the riskiness of each investment – green segments represent the probability that the target VTI will be achieved; the red segments represent the odds of achieving a random VTI somewhere between zero and the target VTI.
